# Posttraumatic arteriovenous fistula of the lower extremity and the pathological characteristic—case series and literature review

**DOI:** 10.1093/jscr/rjad636

**Published:** 2023-12-07

**Authors:** Changhuai He, Pin Ye, Xun Cai, Yiqing Li, Chuanqi Cai

**Affiliations:** Department of General Surgery, Wuhan Asia General Hospital affiliated to Wuhan University of Science and Technology, Wuhan 430056, China; Department of Vascular Surgery, Union Hospital, Tongji Medical College, Huazhong University of Science and Technology, Wuhan 430022, China; Department of Vascular Surgery, Union Hospital, Tongji Medical College, Huazhong University of Science and Technology, Wuhan 430022, China; Department of General Surgery, Wuhan Asia General Hospital affiliated to Wuhan University of Science and Technology, Wuhan 430056, China; Department of Vascular Surgery, Union Hospital, Tongji Medical College, Huazhong University of Science and Technology, Wuhan 430022, China; Department of Vascular Surgery, Union Hospital, Tongji Medical College, Huazhong University of Science and Technology, Wuhan 430022, China

**Keywords:** arteriovenous fistula, posttraumatic fistula, hemodialysis access, pathology, inflow artery, outflow vein, vascular remodeling

## Abstract

The clinical presentation, treatment history, and outcomes of two patients with posttraumatic arteriovenous fistula (PTAVF) were analyzed and compared with the pathological tissues of patients with hemodialysis arteriovenous fistula (HAVF). A search of the biomedical literature database (PubMed), using the keywords “ lower extremity” and “PTAVF,” was conducted to obtain results and review the data. Postoperative histological analysis of patients with PTAVF showed differences from that of HAVF. The literature screening and analysis revealed that PTAVF is a chronic progressive process, with 70% of patients diagnosed after 3 months. The choice of treatment revealed that 20% of patients had severe complications and all were treated endovascularly. Due to the abnormal fistula of PTAVF and its specific histopathological features, the disease is not self-limiting. It is unwise to wait for PTAVF to cause “failure.” We recommend early and timely cure of this disease by surgery to avoid serious complications.

## Introduction

A traumatic arteriovenous fistula (AVF) is a type of vascular injury usually caused by a penetrating injury or crush injury. It has been noted that posttraumatic AVF tends to progress chronically, in contrast to hemodialysis arteriovenous fistula (HAVF). However, there is little pathological evidence to support this hypothesis, and a substantial theoretical basis is lacking. The incidence of posttraumatic AVF is so low that there are not enough cases to form a meaningful statistical analysis of the treatment of posttraumatic AVF, as only 1–2% of trauma patients have a vascular injury, mostly in the lower extremity [1].

## Case 1

**Figure 1:**
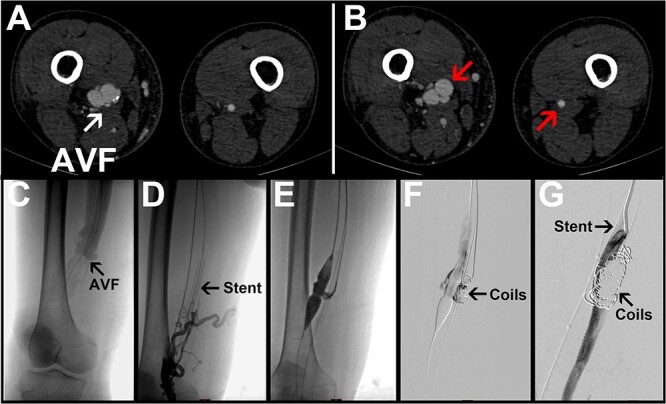
Representative images for Case 1. (A) Preoperative CTA image with the arrow pointing to an arteriovenous fistula. (B) The left arrow points to the obviously dilated right femoral artery; the right arrow points to the normal left femoral artery. (C–G) Intraoperative angiogram images. R, right side; L, left side; AVF, arteriovenous fistula.

A 75-year-old male patient complained of having had bilateral lower limb edema for 1 month. The physical examination revealed sunken edema of both lower limbs, worse on the right side, with a tremor on the right medial thigh. The patient had a previous history of underlying diseases including heart failure, atherosclerosis, hypertension, emphysema, and an NYHA classification of 4 for cardiac function. On admission, a computed tomography angiography (CTA) examination showed a heterogeneous thickening of the lumen of the right femoral artery compared with the left. The right femoral artery was **~14.17** mm in diameter proximally and 5.32 mm in diameter distally; the right femoral abnormal fistula was **~17.28** mm in diameter proximally and 21.01 mm in diameter distally of the femoral fistula. There was a fistula of **~10.25** mm in diameter between the arterioles (Fig. 1A and B). The diagnosis was an AVF. Decades ago, the right inner thigh was struck by a heavy object at work. At that time, there was localized edema, bruising and pain in the inner thigh. Ultrasound and X-ray imaging were performed at the hospital, and there were no abnormalities in the blood vessels and femur. It was not taken seriously attention, and the symptoms were relieved after 2 weeks of rest. There was no history of other trauma. We therefore think that he had an AVF caused by trauma. The patient’s laboratory parameters, computed tomography, and electrocardiogram results were suggestive of abnormal cardiac and pulmonary function. The key to treatment was to improve the circulatory compromise caused by the AVF while maintaining the patient’s basic vital signs. We have developed a variety of treatment options for our patients. Endovascular treatment has less impact on the patient’s circulatory function. However, the patient had atherosclerosis and was prone to embolisms. If the endovascular treatment failed, a surgical repair would be the only remaining option. Through repeated communication with the patient and his family, weighing the pros and cons, the patient opted for endovascular treatment. Under local anesthesia, we introduced a guidewire via the right femoral artery route through the fistula, and one 8 mm × 10 cm Fluency™ stent (BD, Franklin Lakes, NJ) was introduced via the guidewire to be released in the distal segment of the femoral artery. A 16 mm balloon was introduced via the right femoral vein to seal the fistula. The proximal femoral artery was angiographic, and the abnormal tract was not completely closed due to the large diameter of the femoral artery and the continued high-velocity contrast flow that was still present. We then introduced another catheter through the right femoral artery and filled the dilated area of the right femoral artery with 10 and 12 mm coils. The final angiogram showed the satisfactory deployment of the stent and correct exclusion of the fistula tract from both the arterial and venous sides (Fig. 1C–G). Postoperatively, the patient’s cardiopulmonary function did not worsen compared with the preoperative period, the abnormal tremor in the inner thigh disappeared, and the edema of the lower extremity was obviously eliminated. The patient was discharged with instructions to continue long-term treatment with clopidogrel and acetylsalicylic acid. At the 6-month follow-up, the patient complained of gastric discomfort and mild edema in the lower limbs again. The heart failure symptoms were resolved.

## Case 2

**Figure 2:**
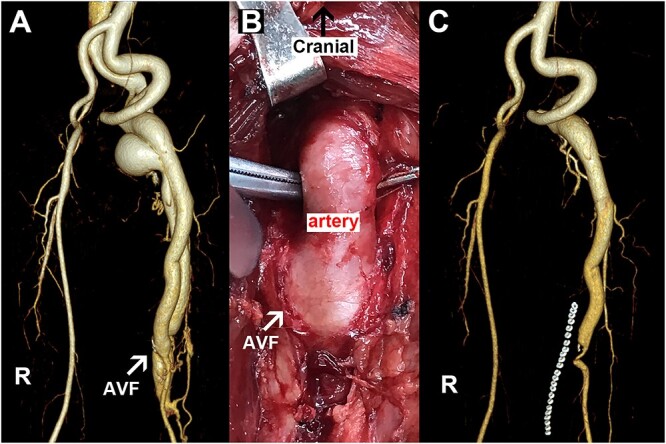
Representative images for Case 2. (A) Preoperative image with the arrow pointing to an AVF. (B) Intraoperative picture with the arrow indicating an AVF. (C) Postoperative imaging data. R, right side; AVF, arteriovenous fistula.

A 53-year-old male patient complained of having had discomfort in his left thigh for 5 months. The physical examination revealed that the patient had edema of the left thigh and local irritation with an abnormal tremor. Forty years earlier, he had been injured by marble on his left thigh. Hospital CT showed soft tissue edema with no femoral or vascular abnormalities, so no attention was taken seriously. The patient’s laboratory tests were unremarkable on admission. Next, the CTA confirmed an abnormal fistula located in the middle of the left femoral artery (Fig. 2A), and the fistula diameter was 12 mm. He was diagnosed with posttraumatic AVF, for which the main treatment modality is surgical excision of the AVF, but there are also newer treatments such as endovascular stenting, embolization, and artificial vascular grafts. In this case, the fistula was a high-flow AVF due to its large size and location in the femoral vessels. Not only was there a risk of multiple endovascular treatments, but the material available for endovascular treatment was not fully adequate for this AVF. We, therefore, recommended him consider the surgical removal rather than endovascular intervention. After assessing the patient’s basic vital signs, we performed the procedure under general anesthesia with an incision in the left medial thigh. As shown in Fig. 2B, the fistula was transected, and the inflow artery and outflow vein were repaired with 6–0 Prolene sutures. Postoperatively, the thrill in the left thigh disappeared immediately. At the end of the procedure, the abnormal tremor in the patient’s inner thighs also disappeared, and the edema in the lower limbs was significantly reduced. On Day 1 post-surgery, the patient was given prophylactic anticoagulant therapy with enoxaparin sodium. Two days later, he was able to walk around without any pain or swelling. Before his discharge, the postoperative CTA scan also confirmed that there were no abnormal postoperative fistulas or venous angiography (Fig. 2C). During the 6-month follow-up, the patient experienced no symptoms or complications.

**Figure 3:**
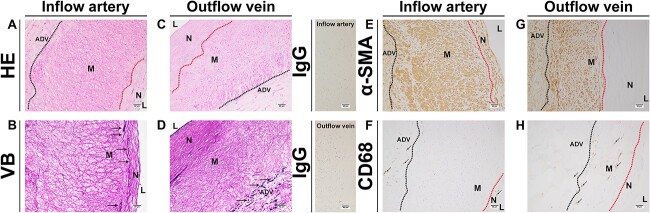
The pathology of the inflow and outflow vessels of the patient in Case 2. (A, C) Representative HE staining of the inflow and outflow vessels. (B, D) Representative Victoria blue staining of the inflow and outflow vessels. (E, G) Representative α-SMA staining of the inflow and outflow vessels. (F, H) Representative CD68 staining of the inflow and outflow vessels. ADV, adventitial layer; M, media layer; N, neointimal layer; α-SMA, α-Smooth muscle actin; VB, Victoria blue staining.

Later, we analyzed the histopathological changes in the vessels adjacent to the AVF in this patient. Firstly, as expected, there was obviously thickened medium in the hematoxylin and eosin (HE) slide of the inflow artery (Fig. 3A), accompanied by broken internal elastic lamina (IEL) (Fig. 3B). There was rare nucleus staining in this posttraumatic AVF (Fig. 3C); positive elastic fiber was only observed in the adventitial layer of this posttraumatic AVF (Fig. 3D). Although there were abundant α-SMA (+) cells in the arterial vessel wall of this posttraumatic AVF (Fig. 3E), positive α-SMA cells were mainly observed in the medium and adventitial layers of the outflow vein (Fig. 3G). There were few CD68 (+) cells in the medium layer of the inflow artery, with clustered CD68 (+) cells in the adventitial and neointimal layers (Fig. 3F). To the contrary, there were positive CD68 cells in the medium layer of the outflow vein but rare positive cells in the neointimal layer (Fig. 3H).

## Discussion

An AVF is a specific form of AVF and is classified as a congenital or acquired fistula. The choice of treatment options, however, varies. It is usually closely related to the general condition of the patients, the level of experience of the surgeons, and the available equipment. It has been reported that endovascular intervention and open surgery are effective modalities for the treatment of posttraumatic AVF [2, 3]. Here, we described two cases of posttraumatic AVF, and selecting the most appropriate treatment plan for each patient. In these cases, we found that the patients had a history of trauma many years prior and had been in a state of chronic progression with no signs of self-limiting closure. In contrast to AVF, there have been many reasons for HAVF failure reported in clinical and experimental studies, including vascular neointimal hyperplasia, hypoxia, inflammation, and epithelial-mesenchymal transition. This interesting phenomenon prompted us to ask: how do posttraumatic AVF and HAVF differ in histopathology? We believe that this report is the first comparison of posttraumatic AVF and HAVF. We analyzed the histopathological characteristics of the inflowing arteries and outflowing veins in Case 2. We found the presence of broken elastic fibers in the inflow and outflow vessels of the posttraumatic AVF. It has been reported that there is excess neointimal hyperplasia with abnormal cellular accumulation in the venous outflow of a HAVF [4]. Similarly, there was obvious neointimal hyperplasia in this posttraumatic AVF. However, in animal AVF models, there is continuous IEL in the outflow vein [4]. But there was rare nucleus staining in this posttraumatic AVF. The positive elastic fiber was only observed in the adventitial layer of this posttraumatic AVF. Positive α-Smooth muscle actin (α-SMA) cells are usually present in the neointimal and medium areas of animal and human nontraumatic AVFs [5]. But there were a few α-SMA-positive cells in the neointimal area in the outflow vein of the posttraumatic AVF, while α-SMA-positive cells are the major cells in HAVF tissues, as we reported elsewhere [4, 5]. Inflammatory cells are proven to be linked to HAVF remodeling and failure [4, 5]. Another important finding was that in the HAVF vessels, there were many inflammatory cells, including CD68(+) cells, while in this posttraumatic AVF sample, both the inflow and outflow vessels presented only scattered CD68(+) cells. Based on these findings, we assume that the posttraumatic AVF is the “matured” AVF compared with the HAVF, and vascular remodeling in a posttraumatic AVF may be distinct to that in a HAVF. This difference may be linked to hemodynamics, as there is usually higher blood flow in a posttraumatic AVF compared with a HAVF.

We searched the literature for posttraumatic AVF cases and summarized the evidence for the different therapeutic strategies to explore the best treatment. With the continuous development of minimally invasive surgery, less invasive and faster recovery has become the goal. However, in the treatment of AVF, endovascular treatment has its limitations. It has been reported that complications of AVF often occur in high-flow vessels [6]. Endovascular treatment of AVF in high-flow thick vessels invariably increases the risk and difficulty of treatment. As shown in Table 1, out of nine patients with femoral vessel injury, 78% (*N* = 7) opted for surgical treatment, and after surgery, the symptoms were effectively relieved; 22% (*N* = 2) opted for endovascular treatment, of which 11% (*N* = 1) required secondary surgical correction due to improper stent deployment and stent misalignment. In the treatment of posttraumatic AVF, we believe that surgery remains the most effective treatment in the absence of contraindications to surgery. Open surgery is also a safe and effective alternative when endovascular treatment fails or complications occur [7]. As shown in Table 1, we classified injuries as “missed” when the time to diagnosis was >3 months, with 70% (*N* = 14) of them presenting with discomfort >3 months after the injury and with no specificity of symptoms, making the diagnosis more difficult. The patient who opted for physiotherapy was found to be progressing at follow-up, with no signs of self-limiting closure [6]. In this regard, we have demonstrated the pathological analysis of patients with posttraumatic AVF, and the results are in stark contrast to patients with HAVF. We reported on a case series and presented its details. This case series may be a useful reference for the future treatment of posttraumatic AVF of the lower extremity. Endovascular treatment is indicated for patients who cannot tolerate open surgery. However, for the average patient, open surgery may be a better option.

**Table 1:** Summary of the findings in reported posttraumatic AVF of the lower extremity cases.

**Author**	**Age (years)**	**Traumatic history**	**Misdiagnosed**	**Location**	**Symptoms**					**Treatments**	**Follow-up visits**
					**Pain**	**Swelling**	**Ulcers**	**Bleeding**	**Heart failure**		
Adeyinka A [6]	16	(+)	(−)	Calf	(+)	(+)	(−)	(−)	(−)	Physiotherapy	Continuous progress
Suknaic S [8]	29	(+)	(+)	Thigh	(−)	(−)	(+)	(−)	(−)	Open surgery	Great
Rehman Z [7]	45	(+)	(+)	Thigh	(+)	(+)	(−)	(−)	(−)	Open surgery	\
Kalender M [9]	52	(+)	(+)	Thigh	(+)	(+)	(−)	(−)	(+)	Open surgery	\
Rehman Z [7]	5.5	(+)	(+)	Thigh	(+)	(−)	(−)	(−)	(−)	Open surgery	\
Siddique M [10]	48	(+)	(+)	Thigh	(+)	(−)	(−)	(−)	(−)	Open surgery	\
Hatch W [11]	33	(+)	(+)	Thigh	(+)	(+)	(+)	(−)	(−)	Open surgery	\
Rehman Z [7]	35	(+)	(+)	Thigh	(−)	(+)	(−)	(−)	(−)	Open surgery	\
Rehman Z [7]	52	(+)	(+)	Knee	(−)	(−)	(+)	(−)	(−)	Open surgery	\
Rehman Z [7]	23	(+)	(−)	Knee	(+)	(+)	(−)	(−)	(−)	Open surgery	\
Rehman Z [7]	30	(+)	(+)	Knee	(−)	(+)	(−)	(−)	(−)	Open surgery	\
Albrecht R [12]	35	(+)	(+)	Calf	(+)	(+)	(−)	(−)	(−)	Open surgery	\
Imai D [13]	69	(+)	(+)	Calf	(−)	(+)	(−)	(−)	(+)	Open surgery	Significant improvement in heart failure
Sahin S [14]	23	(+)	(−)	Thigh	(+)	(+)	(−)	(−)	(−)	Endovascular treatment	Secondary surgical treatment
Rogel-Rodríguez J [15]	16	(+)	(−)	Thigh	(−)	(−)	(−)	(+)	(−)	Endovascular treatment	Long-term anticoagulation
Grandi A [16]	66	(+)	(+)	Knee	(−)	(+)	(−)	(−)	(+)	Endovascular treatment	Indefinite anticoagulation
Rehman Z [7]	17	(+)	(−)	Calf	(−)	(−)	(−)	(+)	(−)	Endovascular treatment	\
Rehman Z [7]	44	(+)	(−)	Calf	(−)	(−)	(−)	(+)	(−)	Endovascular treatment	\
Ray C [17]	47	(+)	(+)	Calf	(−)	(+)	(−)	(−)	(−)	Endovascular treatment	Foot discomfort
Franz R [18]	45	(+)	(+)	Calf	(+)	(+)	(−)	(−)	(−)	Endovascular treatment	Failure, secondary treatment

Misdiagnosed: the definite diagnosis was made 3 months after the symptoms; location: injured area; \: unknown; (+): yes or positive; (−): no or negative.

## Conclusion

Due to the abnormal fistula of posttraumatic AVF and its specific histopathological features, the disease is not self-limiting. It is unwise to wait for posttraumatic AVF to cause “failure.” We recommend early and timely cure of this disease by surgery to avoid serious complications.

## Data Availability

The datasets used and analyzed during the current study are available from the corresponding author on reasonable request.
